# Supplementary material for: Fine control of metal concentrations is necessary for cells to discern zinc from cobalt
Source: Nat Commun. 2017 Dec 1;8:1884. doi: 10.1038/s41467-017-02085-z (PMC5709419; doi:10.1038/s41467-017-02085-z)
Supplement: Supplementary file 1 — Description of Additional Supplementary Files [file 41467_2017_2085_MOESM1_ESM.pdf]

Description of Additional Supplementary Files

File Name: Supplementary Data 1

Description: EXCEL spreadsheet which enables the calculation of DNA occupancy with a DNA-binding protein using Dynafit in conjunction with Supplementary Software

File Name: Supplementary Software

Description: Dynafit script for use with Supplementary Data 1
