# Supplementary material for: Fine control of metal concentrations is necessary for cells to discern zinc from cobalt
Source: Nat Commun. 2017 Dec 1;8:1884. doi: 10.1038/s41467-017-02085-z (PMC5709419; doi:10.1038/s41467-017-02085-z)
Supplement: Supplementary file 2 — Supplementary Information [file 41467_2017_2085_MOESM2_ESM.pdf]

Supplementary Information

Fine control of metal concentrations is necessary for cells to discern zinc from cobalt

Mg(II) < Mn(II) < Fe(II) < Co(II) < Ni(II) < Cu(II) [Cu(I)] > Zn(II)

Supplementary Figure 1| The Irving-Williams series.

The Irving–Williams series (from weak to tight) describes the relative order of metal-association constants, in the absence of steric selection, for essential metals.

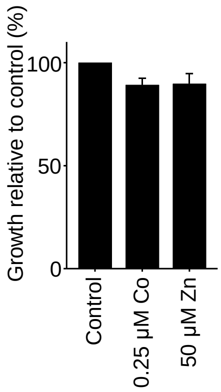

Supplementary Figure 2| Growth during continuous metal exposures shown in Fig. 2.

SL1344^{FrmRE64H} grown to mid-exponential phase in M9 medium (control) and in the presence of 0.25 µM $CoCl_2$ or 50 µM $ZnSO_4$. Values are means of three biological replicates with S.D. (error bars).

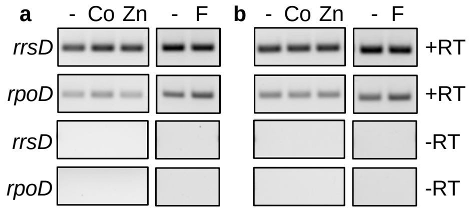

Supplementary Figure 3| Transcript abundance of control genes for Fig. 2.

Transcript abundance (using template generated with and without reverse transcriptase, RT) for control genes *rrsD* (encoding 16S rRNA) and *rpoD* (encoding RNA polymerase σ70) for **Fig. 2** following growth of *Salmonella* in minimal media without supplementation (-), or with

0.25 μM cobalt (Co), 50 μM Zn(II) (Zn) or 50 μM formaldehyde (F). Controls correspond with data for (**a**) Zur, ZntR, RcnR and FrmR^{E64H}-mediated regulation, or (**b**) FrmR-mediated regulation. Full gel images are presented in **Supplementary Figs. 4 and 5**.

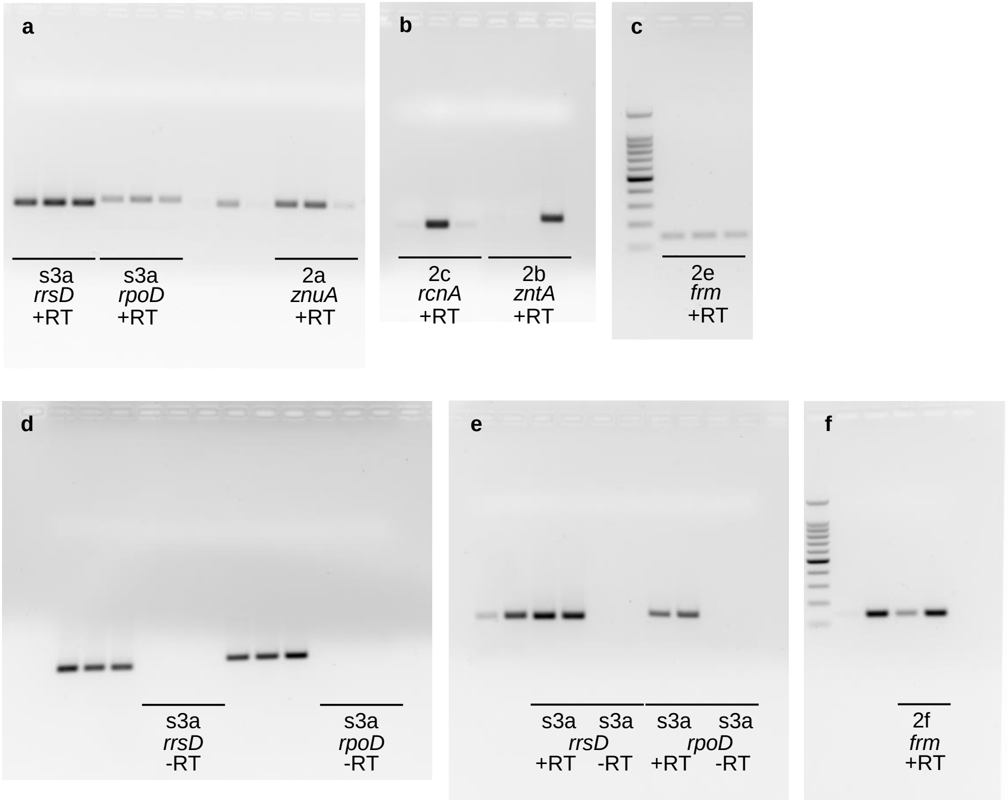

Supplementary Figure 4| Full gel images for Fig. 2 and Supplementary Fig. 3a.

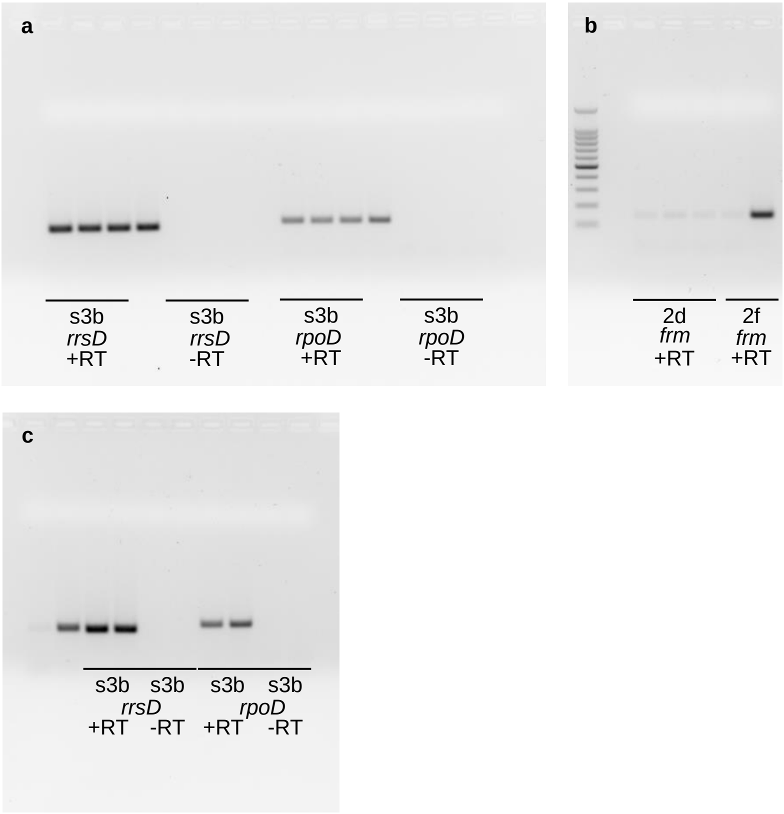

Supplementary Figure 5| Full gel images for Fig. 2 and Supplementary Fig. 3b.

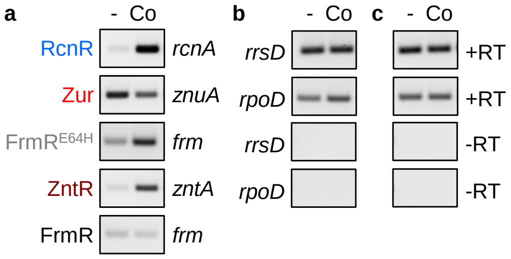

Supplementary Figure 6| Cobalt shock triggered RcnR, Zur, FrmR^{E64H} and ZntR. (**a**) Transcript abundance following 10 min exposure of *Salmonella* without supplementation (-) or to 5 μM $CoCl_2$. (**b,c**) Transcript abundance of control genes *rrsD* and *rpoD* (using template generated with and without reverse transcriptase, RT). Controls correspond with data for (**b**) Zur, ZntR, RcnR and FrmR-mediated regulation, or (**c**) FrmR^{E64H}-mediated regulation. Full gel images are presented in **Supplementary Fig. 7**.

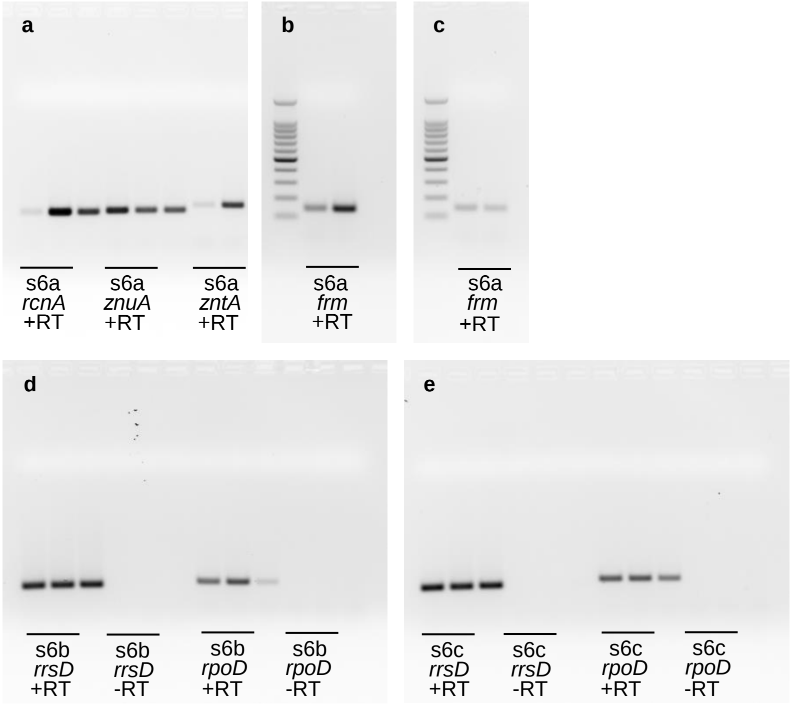

Supplementary Figure 7| Full gel images for Supplementary Fig. 6.

Regulation of *frm* is by $FrmR^{E64H}$ in **b** or FrmR in **c**.

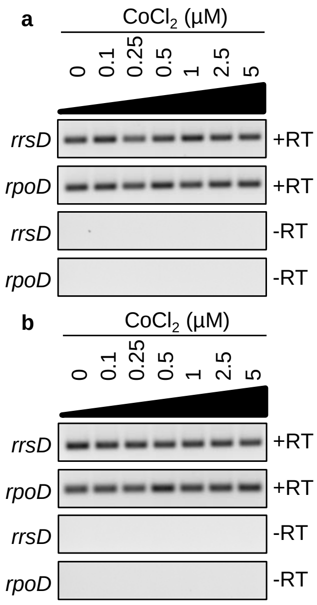

Supplementary Figure 8| Transcript abundance of control genes for Figure 3.

Transcript abundance of control genes *rrsD* and *rpoD* (using template generated with and without reverse transcriptase, RT) for **Fig. 3**. Controls correspond with data for (**a**) Zur, ZntR,

RcnR and FrmR^{E64H}-mediated regulation, or (**b**) FrmR-mediated regulation. Full gel images are presented in **Supplementary Fig. 9**.

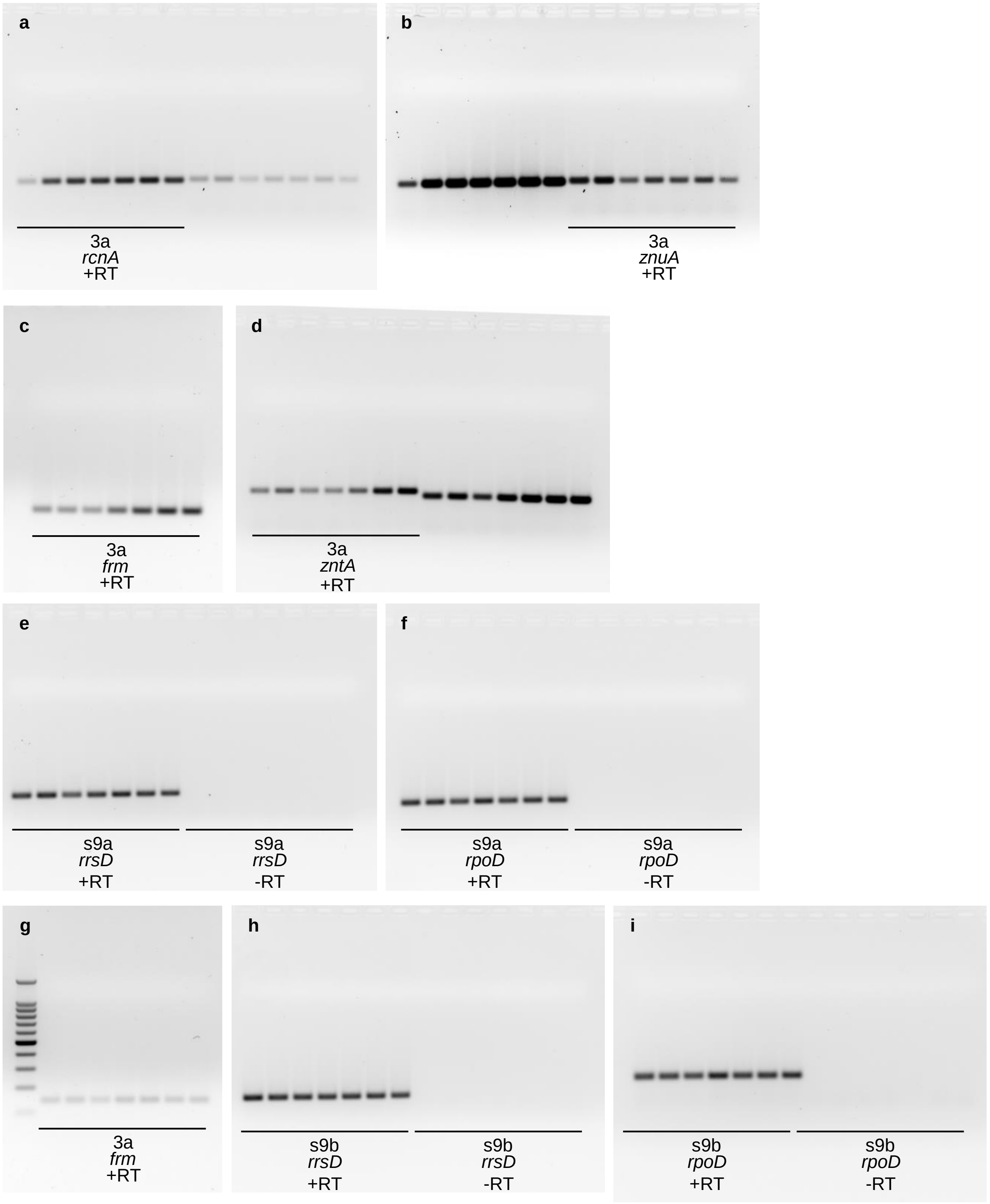

Supplementary Figure 9| Full gel images for Fig. 3 and Supplementary Fig. 8. Regulation of *lacZ* is by FrmR^{E64H} in **c** or FrmR in **g**.

a

$CoCl_2$ (μM)	*rcnA*	*znuA*	*frm*	*zntA*	*frm*
0	1.0 (0.04)	1.0 (0.08)	1.0 (0.10)	1.0 (0.07)	1.0 (0.14)
0.1	5.7 (0.61)	0.9 (0.07)	1.0 (0.04)	1.0 (0.04)	1.1 (0.15)
0.25	36.1 (5.0)	0.5 (0.03)	1.6 (0.17)	1.2 (0.13)	1.2 (0.10)
0.5	57.2 (6.0)	0.5 (0.04)	2.7 (0.19)	1.2 (0.00)	0.9 (0.10)
1	116.3 (27.6)	0.4 (0.06)	11.1 (0.87)	2.8 (0.39)	1.2 (0.05)
2.5	95.4 (13.19)	0.4 (0.06)	21.3 (3.15)	14.0 (1.94)	0.9 (0.15)
5	75.4 (6.17)	0.3 (0.01)	21.6 (1.45)	25.6 (3.79)	0.9 (0.00)
	RcnR	Zur	$FrmR^{E64H}$	ZntR	FrmR

b

$CoCl_2$ (μM)	*rcnA*	*znuA*	*frm*	*zntA*	*frm*
0	1.0 (0.17)	1.0 (0.07)	1.0 (0.15)	1.0 (0.08)	1.0 (0.04)
0.1	1.4 (0.15)	0.9 (0.07)	0.7 (0.03)	1.0 (0.02)	
0.25	2.7 (0.19)	0.8 (0.08)	0.9 (0.14)	1.0 (0.06)	
0.5	7.5 (0.30)	0.9 (0.00)	1.1 (0.05)	2.3 (0.09)	
1	42.4 (4.41)	0.5 (0.05)	2.8 (0.11)	1.1 (0.02)	
2.5	77.2 (6.03)	0.3 (0.03)	4.9 (0.51)	2.8 (0.19)	
5	97.4 (10.1)	0.3 (0.01)	17.2 (1.4)	4.8 (0.28)	0.8 (0.10)
	RcnR	Zur	$FrmR^{E64H}$	ZntR	FrmR

c

$CoCl_2$ (μM)	*rcnA*	*znuA*	*frm*	*zntA*	*frm*
0	1.0 (0.41)	1.0 (0.15)	1.0 (0.27)	1.0 (0.25)	1.0 (0.11)
0.1	7.6 (1.57)	1.0 (0.30)	1.4 (0.19)	0.9 (0.04)	
0.25	32.0 (18.14)	0.5 (0.09)	2.1 (0.40)	1.2 (0.23)	
0.5	62.8 (18.74)	0.3 (0.05)	3.1 (0.00)	1.2 (0.08)	
1	68.1 (14.2)	0.2 (0.11)	19.6 (8.50)	6.1 (1.83)	
2.5	44.2 (27.37)	0.2 (0.07)	27.7 (9.11)	19.3 (5.17)	
5	47.7 (14.41)	0.2 (0.12)	24.4 (3.62)	32.0 (1.30)	1.0 (0.17)
	RcnR	Zur	$FrmR^{E64H}$	ZntR	FrmR

Supplementary Figure 10| Heat maps of gene expression in response to cobalt shock. (**a**) Heat map of qPCR data shown in **Fig. 3**. Values are the fold-change in gene expression relative to the control condition (S.D. in parenthesis). qPCR was performed in triplicate. (**b,c**) Independent culture replicates of **a**. Colours represent the relative change in gene-

expression from lowest to highest: green to yellow for *rcnA*, *frm* and *zntA* and yellow to green for *znuA*. While the absolute response at each cobalt concentration varies across replicates, the sequence of sensor response is preserved (indicated by the similar structure of the heat maps: lower left corner in yellow, which progresses to green in the upper right).

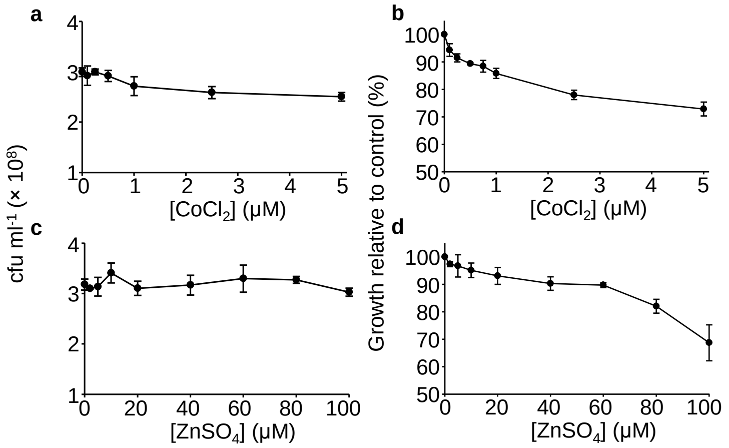

Supplementary Figure 11| Tolerance of *Salmonella* to cobalt and Zn(II).
(**a**) Viability of SL1344^{FrmRE64H} following a 10 min shock of mid-log cells to increasing concentrations of $CoCl_2$ in M9 medium. (**b**) SL1344^{FrmRE64H} grown to mid-exponential phase in M9 medium supplemented with increasing concentrations of $CoCl_2$. (**c**) as '**a**' but with $ZnSO_4$. (**d**). as '**b**' but with $ZnSO_4$. Values are means of three biological replicates with S.D. (error bars).

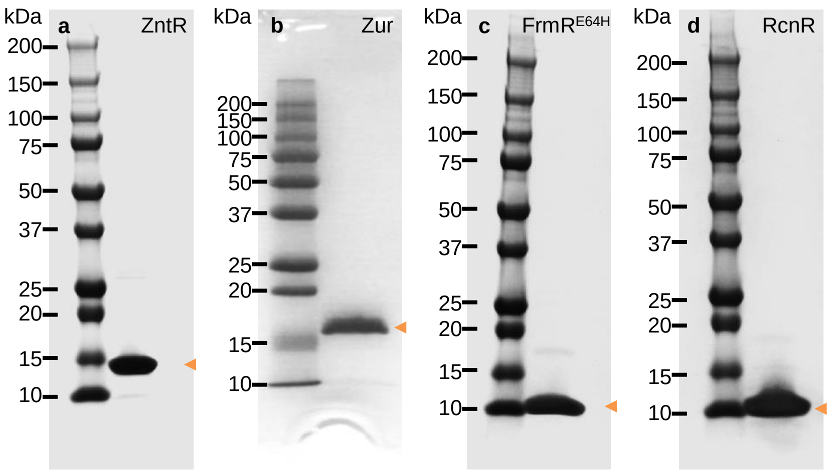

Supplementary Figure 12| Purified ZntR, Zur, FrmR^{E64H} and RcnR.

Analysis of purity of representative preparations of ZntR (**a**), Zur (**b**), FrmR^{E64H} (**c**) and RcnR (**d**). Purified proteins were resolved by SDS-PAGE. Molecular weights are 16.3 kDa for ZntR, 19.2 kDa for Zur, and 10.1 kDa for both FrmR^{E64H} and RcnR. ZntR and Zur migrated with an apparent molecular weight slightly smaller than anticipated, but the sequence and identity of the proteins were confirmed by mass-spectrometry and amino acid analysis.

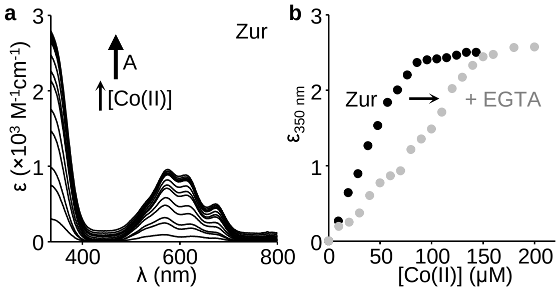

Supplementary Figure 13| Absorption of Co(II)-Zur and competition with EGTA.
(**a**) Zur absorbance spectra upon titration of Zur (50.1 μM) with Co(II). (**b**) Binding isotherm at 350 nm for data shown in **a** (black symbols) and of titration of Zur (50.1 μM) with Co(II) in the presence of EGTA (50 μM) (different data set to **Fig. 4d**).

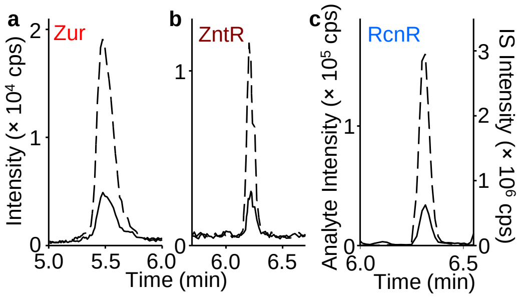

Supplementary Figure 14| Abundance of Zur, ZntR and RcnR.
Representative (n = 3) MRM chromatograms of ion transitions for Zur (**a**), ZntR (**b**), and RcnR (**c**) detected in *Salmonella* cell lysates following growth to exponential phase in minimal media. Transitions are for analyte (solid line) or labelled internal standard (IS, dashed line); cps, counts per second. Data for all experiments are presented in **Supplementary Tables 1-3**.

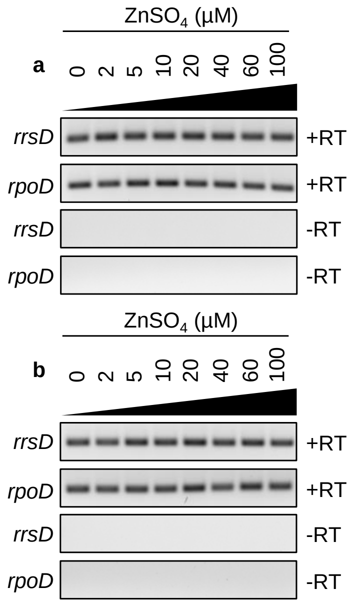

Supplementary Figure 15| Transcript abundance of control genes for Figure 9b. Transcript abundance of control genes *rrsD* and *rpoD* (using template generated with and without reverse transcriptase, RT) for **Fig. 9b**. Controls correspond with data for (**a**) Zur, ZntR, RcnR and $FrmR^{E64H}$-mediated regulation, or (**b**) FrmR-mediated regulation. Full gel images are presented in **Supplementary Fig. 16.**

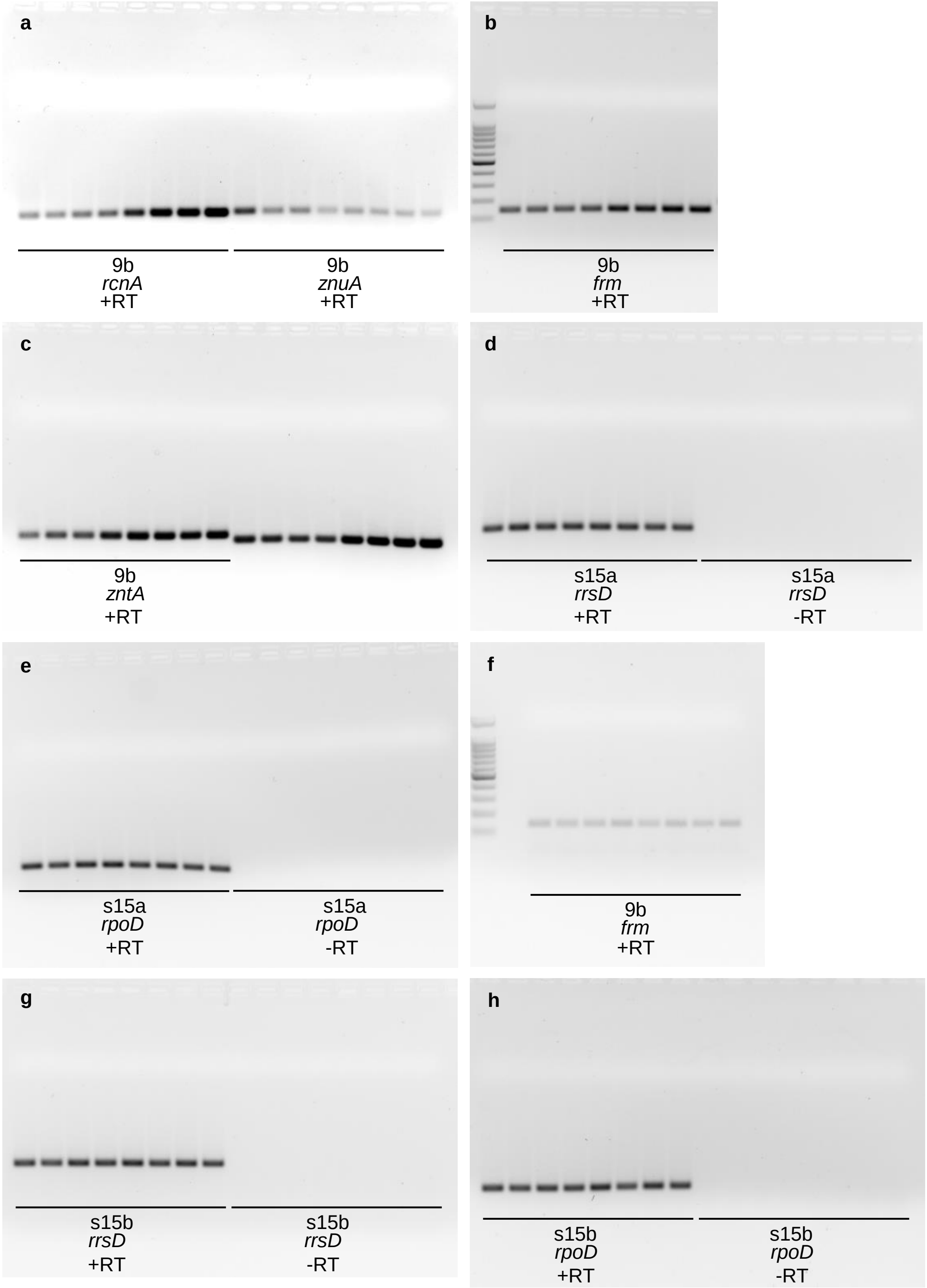

Supplementary Figure 16| Full gel images of Fig. 9b and Supplementary Fig. 15.

Regulation of *frm* is by FrmR^{E64H} in **b** or FrmR in **f**.

a

ZnSO$_4$ (μM)	*znuA*	*zntA*	*frm*	*rcnA*	*frm*
0	1.0 (0.11)	1.0 (0.19)	1.0 (0.14)	1.0 (0.15)	1.0 (0.14)
2	0.7 (0.12)	1.9 (0.20)	1.2 (0.08)	1.0 (0.04)	0.9 (0.20)
5	0.4 (0.04)	1.9 (0.14)	1.3 (0.11)	0.9 (0.11)	1.0 (0.14)
10	0.3 (0.03)	6.1 (0.47)	1.7 (0.12)	1.1 (0.04)	1.4 (0.30)
20	0.3 (0.04)	25.9 (6.12)	6.1 (1.06)	2.5 (0.75)	1.3 (0.14)
40	0.5 (0.07)	70.9 (12.1)	16.5 (2.92)	4.6 (0.48)	1.5 (0.23)
60	0.4 (0.02)	80.8 (6.61)	17.6 (0.71)	5.7 (0.22)	1.5 (0.06)
100	0.4 (0.04)	75.5 (7.85)	17.2 (1.19)	9.8 (1.99)	1.5 (0.16)
	Zur	ZntR	FrmR^{E64H}	RcnR	FrmR

b

ZnSO$_4$ (μM)	*znuA*	*zntA*	*frm*	*rcnA*	*frm*
0	1.0 (0.07)	1.0 (0.11)	1.0 (0.15)	1.0 (0.14)	1.0 (0.10)
4	0.6 (0.09)	5.4 (0.44)	1.1 (0.20)	1.3 (0.14)	
6	0.8 (0.05)	8.8 (0.35)	1.4 (0.11)	2.0 (0.160)	
10	0.5 (0.04)	17.6 (0.69)	2.1 (0.14)	1.8 (0.25)	
40	0.4 (0.017)	165.3 (11.45)	8.2 (0.57)	4.9 (0.00)	
60	0.4 (0.05)	165.3 (11.45)	11.4 (1.18)	5.2 (0.42)	
80	0.5 (0.02)	228.9 (23.80)	14.0 (1.14)	8.4 (0.33)	
100	0.5 (0.07)	252.9 (44.10)	16.4 (1.14)	11.4 (1.41)	1.4 (0.06)
	Zur	ZntR	FrmR^{E64H}	RcnR	FrmR

c

ZnSO$_4$ (μM)	*znuA*	*zntA*	*frm*	*rcnA*	*frm*
0	1.0 (0.10)	1.0 (0.00)	1.0 (0.07)	1.0 (0.11)	1.0 (0.11)
10	0.6 (0.06)	39.6 (4.58)	5.9 (0.62)	3.1 (0.83)	
20	0.5 (0.073)	123.5 (21.82)	13.0 (0.90)	4.1 (0.83)	
40	0.5 (0.06)	233.5 (9.24)	31.3 1.23723	5.3 0.21372	
60	0.4 (0.04)	172.9 (7.00)	18.0 (0.71)	6.7 (0.99)	
80	0.4 (0.06)	308.6 (25.25)	35.1 (1.42)	9.9 (1.66)	
100	0.5 (0.02)	323.8 (34.79)	38.5 (1.52)	14.9 (0.60)	1.3 (0.089)
	Zur	ZntR	FrmR^{E64H}	RcnR	FrmR

Supplementary Figure 17| Heat maps of gene expression in response to Zn(II) shock.

(**a**) Heat map of qPCR data shown in **Fig. 9**. Values are the fold-change in gene expression relative to the control condition (S.D. in parenthesis). qPCRs were performed in triplicate. (**b,c**) Independent culture replicates of **a**. Colours represent the relative change in gene-expression from lowest to highest: green to yellow for *rcnA*, *frm* and *zntA* and yellow to green for *znuA*. While the absolute response at each Zn(II) concentration varies across replicates, the sequence of sensor response is preserved (indicated by the similar structure of the heat maps: lower left corner in yellow, which progresses to green in the upper right).

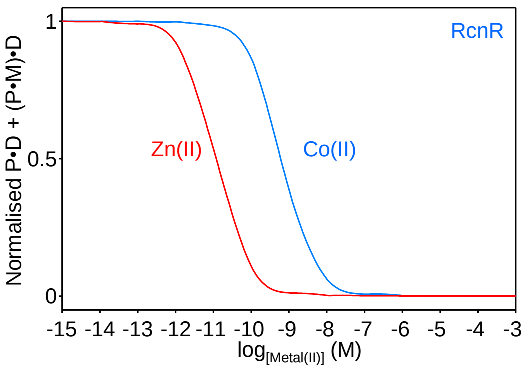

Supplementary Figure 18| Calculated fractional occupancy of DNA with RcnR. Calculated fractional occupancy of DNA target with RcnR as a function of buffered [Zn(II)] (red line) or [Co(II)] (blue line). Data re-plotted from **Fig. 7** and **Fig. 9a** for comparative purposes.

Supplementary Table 1| MRM data for Zur quantitation in cell lysates.

Sample	[Zur] (ng 100 μl^{-1})	Analyte peak area (counts)*	IS peak area (counts)†	Analyte/IS	Calculated [Zur] (ng 100 μl^{-1})‡	Calculated [Zur]/[Zur]
**STD 1**	2.5	1.24E+04	2.36E+05	0.05	1.93	0.77
**STD 2**	5	2.89E+04	2.17E+05	0.13	5.02	1.00
**STD 3**	10	6.12E+04	2.19E+05	0.28	10.7	1.07
**STD 4**	50	2.57E+05	2.22E+05	1.16	45.6	0.91
**STD 5**	150	7.20E+05	2.12E+05	3.40	148	0.99
**STD 6**	200	9.63E+05	2.07E+05	4.65	217	1.09
**QC**	7	4.26E+04	2.18E+05	0.20	7.4	1.06
**M9 1**	n.a.	7.99E+04	2.24E+05	0.36	13.7	n.a.
**M9 2**	n.a.	7.34E+04	2.01E+05	0.37	14	n.a.
**M9 3**	n.a.	7.37E+04	2.07E+05	0.36	13.7	n.a.
**QC**	7	4.63E+04	2.26E+05	0.20	7.8	1.11
**STD 1**	2.5	1.62E+04	2.11E+05	0.08	2.86	1.14
**STD 2**	5	2.95E+04	2.00E+05	0.15	5.56	1.11
**STD 3**	10	5.72E+04	2.02E+05	0.28	10.8	1.08
**STD 4**	50	2.27E+05	2.07E+05	1.10	43.3	0.87
**STD 5**	150	6.68E+05	2.05E+05	3.26	141	0.94
**STD 6**	200	8.63E+05	1.91E+05	4.52	209	1.05

n.a. = not applicable. STD 1-6 = known concentrations of Zur which make up a front and back standard curve. QC = quality control sample of known concentration. M9 1-3 = independent cell lysate preparations of *Salmonella* SL1344 (n = 3) grown in M9 media.

*Analyte peptide is ETEPQAKPPTIYR.

†Internal standard (IS) is labelled ETEPQAKPPTIYR[$^{13}C_6$, $^{15}N_4$].

‡Calculated using the two standard curves shown using a quadratic 1/x^2 weighted regression model.

Supplementary Table 2| MRM data for ZntR quantitation in cell lysates.

Sample	[ZntR] (ng 100 μl^{-1})	Analyte peak area (counts)*	IS peak area (counts)†	Analyte/IS	Calculated [ZntR] (ng 100 μl^{-1})‡	Calculated [ZntR]/[ZntR]
**STD 1**	2.5	2.84E+03	4.97E+04	0.06	2.4	0.96
**STD 2**	5	4.93E+03	4.84E+04	0.10	5.2	1.04
**STD 3**	10	8.45E+03	4.67E+04	0.18	10.1	1.01
**STD 4**	50	3.48E+04	4.71E+04	0.74	45	0.90
**STD 5**	150	1.04E+05	4.35E+04	2.39	146	0.97
**STD 6**	200	1.40E+05	4.19E+04	3.34	203	1.02
**QC**	7	5.56E+03	4.43E+04	0.13	6.9	0.99
**M9 1**	n.a.	1.55E+04	4.61E+04	0.34	19.8	n.a.
**M9 2**	n.a.	1.40E+04	4.34E+04	0.32	19	n.a.
**M9 3**	n.a.	1.34E+04	4.98E+04	0.27	15.6	n.a.
**QC**	7	7.68E+03	5.13E+04	0.15	8.2	1.17
**STD 1**	2.5	3.00E+03	5.07E+04	0.06	2.53	1.01
**STD 2**	5	4.13E+03	4.26E+04	0.10	4.89	0.98
**STD 3**	10	8.26E+03	4.35E+04	0.19	10.7	1.07
**STD 4**	50	3.52E+04	4.28E+04	0.82	50.1	1.00
**STD 5**	150	1.04E+05	4.13E+04	2.52	155	1.03
**STD 6**	200	1.36E+05	4.15E+04	3.28	200	1.00

n.a. = not applicable. STD 1-6 = known concentrations of ZntR which make up a front and back standard curve. QC = quality control sample of known concentration. M9 1-3 = independent cell lysate preparations of *Salmonella* SL1344 (n = 3) grown in M9 media.

*Analyte peak is LADVTPDTIR.

†Internal standard (IS) is labelled LADVTPDTIR[$^{13}C_6$, $^{15}N_4$].

‡Calculated using the two standard curves shown using a quadratic $1/x^2$ weighted regression model.

Supplementary Table 3| MRM data for RcnR quantitation in cell lysates.

Sample	[RcnR] (ng 100 µl^{-1})	Analyte peak area (counts)*	IS peak area (counts)†	Analyte/IS	Calculated [RcnR] (ng 100 µl^{-1})‡	Calculated [RcnR]/[RcnR]
**STD 1**	5	8.03E+04	1.25E+07	0.006	4.92	0.98
**STD 2**	10	1.33E+05	1.23E+07	0.011	9.12	0.91
**STD 3**	20	3.01E+05	1.31E+07	0.023	20.7	1.04
**STD 4**	50	6.17E+05	1.21E+07	0.051	46.1	0.92
**STD 5**	100	1.31E+06	1.23E+07	0.107	93.5	0.94
**STD 6**	200	2.99E+06	1.16E+07	0.258	210	1.05
**QC**	7.5	9.69E+04	1.25E+07	0.008	6.22	0.83
**M9 1**	n.a.	1.16E+05	1.23E+07	0.009	7.82	n.a.
**M9 2**	n.a.	9.58E+04	1.16E+07	0.008	6.72	n.a.
**M9 3**	n.a.	1.22E+05	1.12E+07	0.011	9.26	n.a.
**QC**	7.5	8.64E+04	1.11E+07	0.008	6.22	0.83
**STD 1**	5	7.34E+04	1.17E+07	0.006	4.81	0.96
**STD 2**	10	1.51E+05	1.07E+07	0.014	12.3	1.23
**STD 3**	20	2.27E+05	1.12E+07	0.020	18	0.90
**STD 4**	50	6.47E+05	1.13E+07	0.057	51.6	1.03
**STD 5**	100	1.30E+06	1.06E+07	0.123	108	1.08
**STD 6**	200	2.43E+06	1.05E+07	0.231	191	0.96

n.a. = not applicable. STD 1-6 = known concentrations of RcnR which make up a front and back standard curve. QC = quality control sample of known concentration. M9 1-3 are independent cell lysate preparations of *Salmonella* SL1344 (n = 3) grown in M9 media.

*Analyte peak is GAVNGLMR.

†Internal standard (IS) is labelled GAVNGLM<u>R</u>[$^{13}C_6$, $^{15}N_4$].

‡Calculated using the two standard curves shown using a quadratic 1/x^2 weighted regression model.

Supplementary Table 4| Oligonucleotides used in this study.

No.	Primer name	Sequence	Source
**1**	Ec_zur_del_F	5′-AACATGGTAAAGTAAGGACATTCTTAACCCCCACTTTGAGGTGCCCGATG-3′	This study
**2**	Ec_zur_del_R	5′-GCCCGACGTGTACAAGGATGTACGCCCTCTTAACGCGGTTTCTTTTTCAC-3′	This study
**3**	Ec_zur_F	5′-GGCAACAATAAGGGTTCTCG-3′	This study
**4**	Ec_zur_R	5′-GAAACCCGCAATGAATATCG-3′	This study
**5**	Ec_zntR_F	5′-GCAACGGAGAGAAGCAATTC-3′	This study
**6**	Ec_zntR_R	5′-GTTATTTAACGGCGCGAGTG-3′	This study
**7**	Ec_rcnR_F	5′-AAGCGCACCAAGTAAGATGG-3′	This study
**8**	Ec_rcnR_R	5′-TTCCCTACGCTGGCATTATC-3′	This study
**9**	Ec_nikR_F	5′-AACGGACAAATCGTCGAAAC-3′	This study
**10**	Ec_nikR_R	5′-TGTTGATCGTTCACCAAAGG-3′	This study
**11**	rcnA_RTPCR_F	5′-TCCCAGCGCCATTTTATTAG-3′	This study
**12**	rcnA_RTPCR_R	5′-ACGATCGCGGTATGAGAAAG-3′	This study
**13**	znuA_RTPCR_F	5′-ACATGCATCTTTGGCTCTCC-3′	This study
**14**	znuA_RTPCR_R	5′-ACCGACCTGTTTATCGGTTG-3′	This study
**15**	zntA_RTPCR_F	5′-TAAACTGGTTTCCGGTTTCG-3′	This study
**16**	zntA_RTPCR_R	5′-TAAACTGGTTTCCGGTTTCG-3′	This study
**17**	lacZ_RTPCR_F	5′-ACGGGTTGTTACTCGCTCAC-3′	This study
**18**	lacZ_RTPCR_R	5′-AAATTCAGACGGCAAACGAC-3′	This study
**19**	rrsD_RTPCR_F	5′-GCACAACCTCCAAGTAGACATC-3′	This study
**20**	rrsD_RTPCR_R	5′-GGTGAAATGCGTAGAGATCTGG-3′	This study
**21**	rpoD_RTPCR_F	5′-CAACCGTATTTCTCGCCAGATG-3′	This study
**22**	rpoD_RTPCR_R	5′-CACCCAGATGCGAATCTTCATC-3′	This study
**23**	znuAPro_F	5′-[HEX]TAGAATGTTATAATATCACATTTCACACATTCA-3′	This study
**24**	znuAPro_R	5′-TGAATGTGTGAAATGTGATATTATAACATTCTA-3′	This study
**25**	zntAPro_F	5′-[HEX]CTTGACTCTGGAGTCGACTCCAGAGTGTATCCTC-3′	This study
**26**	zntAPro_R	5′-GAGGATACACTCTGGAGTCGACTCCAGAGTCAAG-3′	This study
**27**	rcnAPro_F	5′-[HEX]TACTCCCCCCCAGTATAGAATACTACCCCCCAGTA-3′	(1)
**28**	rcnAPro_F	5′-TACTGGGGGGTAGTATTCTATACTGGGGGGGAGTA-3′	(1)
**29**	frmRPro_F	5′-[HEX]TTCTGATAGTATACCCCCCTATAGTATATGGAG-3′	(2)
**30**	frmRPro_R	5′-CTCCATATACTATAGGGGGGTATACTATCAGAA-3′	(2)

Supplementary References.

1. Osman, D. *et al*. The effectors and sensory sites of formaldehyde-responsive regulator FrmR and metal-sensing variant. *J. Biol. Chem*. **291**, 19502-19516 (2016).
2. Osman, D. *et al*. Generating a metal-responsive transcriptional regulator to test what confers metal sensing in cells. *J. Biol. Chem*. **290**, 19806-19822 (2015).
